# Monitoring Response to Transarterial Chemoembolization in Hepatocellular Carcinoma Using ^18^F-Fluorothymidine PET

**DOI:** 10.2967/jnumed.119.240598

**Published:** 2020-12

**Authors:** Rohini Sharma, Marianna Inglese, Suraiya Dubash, Haonan Lu, David J. Pinato, Chandan Sanghera, Neva Patel, Anthony Chung, Paul D. Tait, Francesco Mauri, William R. Crum, Tara D. Barwick, Eric O. Aboagye

**Affiliations:** 1 Department of Surgery and Cancer, Imperial College London, Hammersmith Hospital, London, United Kingdom; 2 Radiological Sciences Unit, Imperial College Healthcare NHS Trust, London, United Kingdom; 3 Department of Radiology, Imperial College Healthcare NHS Trust, London, United Kingdom; and; 4 Institute of Translational Medicine and Therapeutics, Imperial College London, London, United Kingdom

**Keywords:** ^18^F-FLT PET, hepatocellular cancer, response

## Abstract

Accurate disease monitoring is essential after transarterial chemoembolization (TACE) in hepatocellular carcinoma (HCC) because of the potential for profound adverse events and large variations in survival outcome. Posttreatment changes on conventional imaging can confound determination of residual or recurrent disease, magnifying the clinical challenge. On the basis of increased expression of thymidylate synthase (*TYMS*), thymidine kinase 1 (*TK-1*), and equilibrative nucleoside transporter 1 (*SLC29A1*) in HCC compared with liver tissue, we conducted a proof-of-concept study evaluating the efficacy of 3′-deoxy-3′-^18^F-fluorothymidine (^18^F-FLT) PET to assess response to TACE. Because previous PET studies in HCC have been hampered by high background liver signal, we investigated whether a temporal-intensity voxel clustering (kinetic spatial filtering, or KSF) improved lesion detection. **Methods:** A tissue microarray was built from 36 HCC samples and from matching surrounding cirrhotic tissue and was stained for *TK-1*. A prospective study was conducted; 18 patients with a diagnosis of HCC by the criteria of the American Association for the Study of Liver Diseases who were eligible for treatment with TACE were enrolled. The patients underwent baseline conventional imaging and dynamic ^18^F-FLT PET with KSF followed by TACE. Imaging was repeated 6–8 wk after TACE. The PET parameters were compared with modified enhancement-based RECIST. **Results:** Cancer Genome Atlas analysis revealed increased RNA expression of *TYMS, TK-1,* and *SLC29A1* in HCC. *TK-1* protein expression was significantly higher in HCC (*P* < 0.05). The sensitivity of ^18^F-FLT PET for baseline HCC detection was 73% (SUV_max_, 9.7 ± 3.0; tumor to liver ratio, 1.2 ± 0.3). Application of KSF did not improve lesion detection. Lesion response after TACE by modified RECIST was 58% (14 patients with 24 lesions). A 30% reduction in mean ^18^F-FLT PET uptake was observed after TACE, correlating with an observed PET response of 60% (15/25). A significant and profound reduction in the radiotracer delivery parameter *K*_1_ after TACE was observed. **Conclusion:** ^18^F-FLT PET can differentiate HCC from surrounding cirrhotic tissue, with PET parameters correlating with TACE response. KSF did not improve visualization of tumor lesions. These findings warrant further investigation.

The recommended treatment option for intermediate-stage hepatocellular carcinoma (HCC) is transarterial chemoembolization (TACE), which involves the delivery of a cytotoxic agent commonly mixed with lipiodol followed by selective embolization of the tumoral arterial supply (1). The typical vascular pattern of HCC on contrast-enhanced CT or MRI is early arterial enhancement followed by washout. Although both contrast-enhanced CT and MRI are widely used to assess response after TACE, there is uncertainty in their ability to detect viable disease after TACE (2). Modified RECIST (mRECIST), which measures changes in arterial enhancement as a marker of residual viable tumor, is a more accurate measure of tumor response to treatment than standard RECIST and is routinely used in the assessment of HCC (3). However, lipiodol deposition can induce beam-hardening artifacts and obscure enhancement in the arterial phase, reducing the sensitivity of CT after TACE. With MRI, coagulative hemorrhagica necrosis may lead to a high T1 signal, making it difficult to assess enhancement (4).

PET imaging has been evaluated in HCC for staging and response assessment (5). Studies investigating ^18^F-FDG in HCC show limited sensitivity (50%–70%) due to similar activities of glycolytic enzymes and glucose 6-phosphatase in liver and well-differentiated HCC, resulting in near-equivalent uptake of ^18^F-FDG (6). In imaging with single-agent ^11^C-acetate and ^11^C-choline, ^18^F-choline are similarly limited, culminating in the exploitation of dual-tracer techniques to improve sensitivity and specificity (7).

3′-deoxy-3′-^18^F-fluorothymidine (^18^F-FLT) is a surrogate marker of proliferation, with uptake reflecting the activity of thymidine kinase 1 (*TK-1*), whose expression correlates with ex vivo proliferation biomarkers (8). Unlike ^18^F-FDG, the uptake of ^18^F-FLT is more specific for proliferation and is unaffected by inflammation, a particular concern because HCC tumors develop within a proinflammatory milieu (9). To date, a single study of ^18^F-FLT PET in HCC indicated that 69% of patients had uptake higher than background liver whereas the remaining lesions were either photopenic or of mixed uptake (10). However, the patient group was heterogeneous, including cholangiocarcinoma, and no information was given about therapy response. To improve lesion detection, we have previously applied a temporal voxel-clustering approach—kinetic spatial filtering (KSF)—for removing normal, physiologic hepatic ^18^F-FLT uptake and to visualize specific uptake (i.e., uptake due to phosphorylation) in liver metastases (11). Briefly, the KSF compares the time–activity curves of each voxel with the time–activity curve of predefined tissue classes such as liver and tumor. Voxels classed as liverlike are excluded, thereby removing areas of physiologic uptake unrelated to ^18^F-FLT retention.

This study evaluated the clinical utility of ^18^F-FLT PET in assessing TACE response in HCC. We first reviewed the RNA expression of key targets in the metabolism of ^18^F-FLT using large published datasets of HCC. We then investigated the tissue expression of *TK-1* in HCC and surrounding cirrhosis, an important consideration in developing a tracer paradigm that will effectively differentiate cirrhotic tissue from HCC. Finally, we undertook a prospective study using dynamic ^18^F-FLT PET both to visualize the tumors and to use as a response biomarker, incorporating application of the KSF and kinetic modeling.

## MATERIALS AND METHODS

### Bioinformatics Analysis

An RNA-sequencing dataset containing 371 HCC and 50 nonmalignant tissue samples from The Cancer Genome Atlas project was measured from the Illumina HiSEquation 2000 RNA Sequencing platform. The RNA-Seq by expectation maximization–normalized data were downloaded from UCSC Xena (http://xena.ucsc.edu/). Differential gene expression of *TK1,* thymidylate synthase (*TYMS*), and equilibrative nucleoside transporter 1 (*SLC29A1*) comparing tumor and nonmalignant tissue was performed using the ggplot2 package and the t.test function in R, version 3.5.2.

### Tissue Microarray

Immunohistochemistry for *TK-1* (1:100; AbCam) was performed on a tissue microarray from 36 patients with a histologic diagnosis of HCC. A trained histopathologist who was unaware of the clinical data scored all cases manually using the immunohistochemical score (12). Access to retrospective tissue specimens was granted by the Imperial College Tissue Bank (approval R16005).

### Prospective Study Design

Eighteen patients with HCC were prospectively enrolled (Supplemental Appendix 1; supplemental materials are available at http://jnm.snmjournals.org). Patients received standard TACE with liposomal doxorubicin emulsified in lipiodol followed by embolization with gelatin sponge particles. Baseline staging included contrast-enhanced CT or MRI of the liver 28 d before TACE; the same imaging modality was repeated 6–8 wk after TACE to evaluate treatment response, followed by 3 monthly until disease progression. mRECIST for HCC (13) was documented by a single experienced hepatobiliary radiologist.

### Image Analysis

Lesions on ^18^F-FLT PET corresponding to those larger than 10 mm on CT or MRI and showing increased uptake were considered target lesions and used for analyses both on PET/CT and on CT or MRI before and after treatment.

Consecutive regions of interest were manually defined on the summed images and encompassed the whole tumor for SUV analysis. The ^18^F-FLT radioactivity concentration within the regions of interest was normalized for injected radioactivity and body weight (grams) to obtain SUV_mean_ and SUV_max_ at 60 min (SUV60_mean_ and SUV60_max_, respectively) on baseline and posttreatment ^18^F-FLT PET/CT studies. The percentage change in both SUV_mean_ and SUV_max_ was calculated for each target lesion visible on baseline imaging as (posttreatment SUV – baseline SUV)/baseline SUV. In each case, a 3-cm region of interest was placed in the liver in a tumor-free area to measure background liver SUV_mean_, and the ratio of tumor SUV_max_ to liver SUV_mean_ was determined.

### Quantitative Analysis

A metabolite-corrected image-derived arterial input function was im-plemented during kinetic modeling of data with a 2-tissue-compartment model.

### Statistical Analysis

Because this was a pilot study, no formal power calculation was undertaken. Summary statistics of the associations between PET parameters and clinical outcome were determined. Because of the small sample size, patients were grouped as responders (complete or partial response) or nonresponders (stable or progressive disease). The relationship between kinetic parameters and response was evaluated using Wilcoxon rank tests. The χ^2^ test was used to evaluate the utility of the tracer before and after TACE therapy. Concordance was determined using Cohen κ-analysis. A *P* value of 0.05 or less was considered significant. All statistical analyses were conducted using the SPSS statistical package, version 22 (SPSS Inc.).

## RESULTS

### Increased Expression of Thymidine Metabolism Enzymes in HCC

Using RNA-sequencing data from The Cancer Genome Atlas, we observed a significantly higher expression of *TYMS, TK-1,* and *SLC29A1* in tumor tissue (*n* = 371) than in adjacent nonmalignant tissue (*n* = 50; *P* < 0.05) (Supplemental Figs. 1A–1C). Using a tissue microarray, we observed a significantly higher *TK-1* expression in HCC tumors (median immunohistochemical score, 33; range, 0–300) than in the surrounding parenchyma (score, 0; range, 0–50), suggesting that ^18^F-FLT has the potential to differentiate HCC from surrounding cirrhotic liver (*P* = 0.004) (Supplemental Figs. 2A–2C).

### Patient Characteristics

Eighteen patients were enrolled (16 men and 2 women), with 16 patients completing the study (Table 1). The median age was 68 y (range, 42–79 y). All patients received TACE for intermediate-stage disease. Three patients had received TACE previously and were undergoing retreatment for residual active disease; the remaining patients were treatment-naïve.

**TABLE 1:** Imaging Features of HCC Lesions

Patient no.	Lesion location	Lesion size (mm)	Uptake above background (visual)	Background SUV_mean_	SUV60 × 10^−5^m^2^mL^−1^ before Rx		mRECIST response	Percentage change in SUV60		Progression- free survival (mo)
					Mean	Maximum		Mean	Maximum	
1	Diffuse disease, R lobe	117	Isotense	4.3	3.4	5.1	PR	−12.2	13.9	3
2	Segment V/VI	56	Hyperintense rim and hypointense center	4.9	4.3	7.8	PR	−55.5	−45.5	6.6
3	Segment VII	60	Isotense	6.3	5.7	7.9	SD	−65.2	−42.6	5.3
4	Segment VI	20	Isotense	5.5	2.8	5.5	SD	−6.0	−14.4	4.5
4	Segment V	52	Isotense	5.5	4.1	5.8	SD	−0.9	−6.7	
5	Segment II	44	Hyperintense	6.6	7.5	10.3	SD	−37.4	1.4	11.4
	Segment VI*	24	Hyperintense	6.6	7.6	9.9	SD	10.9	−0.7	
	Segment IV	19	Hyperintense	6.6	7.3	9.8	SD	15.5	26.2	
	Segment IV	16	Hyperintense	6.6	7.8	10.3	SD	5.9	1.9	
	Segment VIII	15	Hyperintense	6.6	7.1	9.4	SD	0.07	0.9	
	Segment IV	13	Hyperintense	6.6	7.8	11.3	SD	−4.7	0.9	
	Segment VIII	19	Hyperintense	6.6	7.1	8.2	PR	22.2	36.5	
6	Segment III	22	Hyperintense	4.8	7.6	10.2	NE	−69.2	−61.9	17.3
7	Segment III	22	Hyperintense	6.1	10.9	14.3	NE	NE	NE	8.0
8	Segment VIII	42	Hyperintense	6.5	7.8	11.5	PR	−66.4	−41.5	1.5
9	Segment II	28	Hyperintense	6.3	7.9	10.3	CR	−58.6	−46.1	5.2
10	Segment I	62	Hyperintense	7.7	8.1	20.4	PR	−71.3	−41.0	1.1^†^
11	Segment III	38	Hyperintense	6.1	7.1	10.6	PR	−29.0	−21.5	7.8
12	Segment VIII	59	Mixed isotense and hypotense	8.0	5.1	10.9	PR	−55.8	−32.8	16.7
12	Segment IV^‡^	60	Hypotense	8.0	2.9	8.4	PR	−51.6	−33.9	
13	Segment VI	31	Hyperintense	4.8	6.4	8.9	CR	−39.4	−48.6	19.1
14	Segment VII (sagittal)	18	Hyperintense	5.3	5.3	7.4	CR	−33.9	−30.5	8.1
14	Segment VIII	26	Hyperintense	5.3	6.3	7.5	CR	−33.5	−39.1	
14	Segment VII (medial)	10	Hyperintense	5.3	6.6	8.0	SD	17.2	30.9	
15	Segment VIII	34	Hyperintense	5.5	7.3	10.4	PR	−52.4	−46.3	8.9
16	Segment VI/VII	73	Hyperintense	5.7	6.6	11.6	PR	−65.8	−24.1	14.5^¶^

* Untreated lesion.

† Patient died of unrelated illness.

‡ Photopenic lesion.

¶ Patient underwent liver transplantation.

Rx = treatment; PR = partial response; SD = stable disease; NE = not evaluable; CR = complete response.

### Visibility of HCC Above Background on ^18^F-FLT PET Images

**FIGURE 1.**
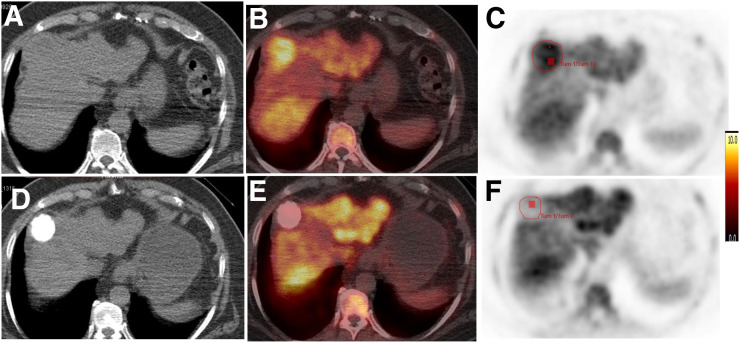
Axial CT, ^18^F-FLT PET/CT, and ^18^F-FLT PET images before (A–C) and after (D–F) TACE show focal HCC lesion (red outline) with increased uptake at baseline and reduction after TACE. Tum = tumor.

Twenty-six liver lesions (median size, 29.5 mm; range, 10–117 mm) were identified on conventional imaging; 5 had baseline MRI and the remainder CT. On visual analysis of the PET images, 19 lesions had a level of uptake above that of the background liver (73% sensitivity) (Figs. 1A–1F).

All lesions were included in the analysis. The mean SUV60_mean_ (±SD) and SUV60_max_ on baseline imaging were 6.5 ± 1.9 and 9.7 ± 3.0, respectively. The SUV60_mean_ of the background liver was 6.1 ± 0.9. A significant difference was observed between the SUV60_max_ of the cancer and that of the surrounding, noncancerous, liver tissue (*P* = 0.02), with a tumor-to-liver ratio of 1.2 ± 0.3, confirming that uptake in HCC was above cirrhotic background activity, enabling visualization on ^18^F-FLT PET scans in most cases.

### No Improvement of Visualization upon KSF

**FIGURE 2.**
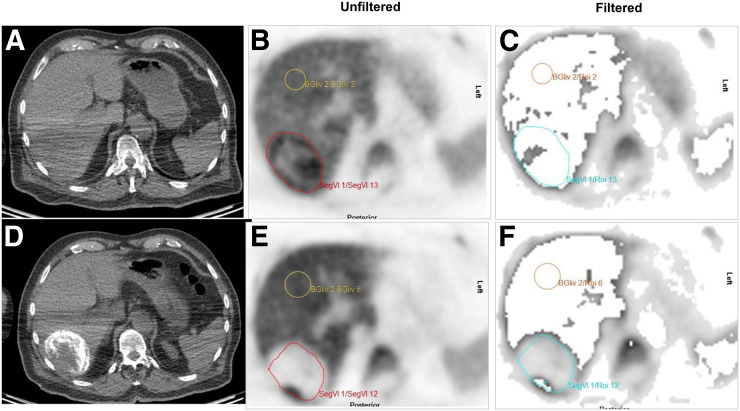
Axial CT, unfiltered ^18^F-FLT PET, and filtered ^18^F-FLT PET images before (A–C) and after (D–F) TACE show focal HCC lesion (red outline) with increased uptake at baseline and reduction after TACE. (B and E) In one of two baseline PET images before application of KSF (B), HCC is visible above background. (C) After application of KSF, tumor is mostly filtered out. (E) In post-TACE images, HCC is photopenic compared with surrounding liver before application of KSF. (F) After application, background liver activity is removed and HCC remains visible. BGliv = background liver; Seg = segment. Yellow outline indicates background liver, and blue outline indicates focal HCC lesion on following application of KSF.

Background liver activity was completely filtered out in 12 of 16 patients; 4 patients retained partial background liver activity. KSF did not improve image visualization above that of PET/CT imaging; 11 of the 26 lesions (42%) were visible after application of KSF, compared with 19 lesions without KSF (Figs. 2A–2F). Small lesions typically had a homogeneous appearance, whereas larger lesions were characterized by perilesional tracer uptake with no measurable ^18^F-FLT trapping in the necrotic center of the tumor. Of the 15 lesions not visualized through KSF, 9 (60%) were smaller than 30 mm; 3 lesions had higher tissue activity than the HCC average predetermined by the KSF, and the remaining lesions did not retain radiotracer after application of the KSF. Because KSF is associated with removal of delivery components within the data, there was a mean signal reduction of 81% in the tumors at baseline (range, 18%–100%) relative to the unfiltered images. The mean reduction in background activity in the liver was 98% (range, 83%–100%), resulting in an improved tumor-to-liver ratio of 11.1 ± 17.7.

### ^18^F-FLT Uptake Parameters and Clinical Outcome

In terms of response to TACE according to mRECIST, 24 lesions were assessable and 2 were not (1 patient withdrew consent after baseline PET). A response was observed in 14 of the 24 lesions (58%); no response was observed in the other 10 (42%). There was a median overall reduction in SUV60_mean_ (−29.5% ± 31.4%) and SUV60_max_ (−18.5% ± 27.5%) after TACE. Previous test–retest reproducibility studies in breast cancer considered changes in ^18^F-FLT SUV of more than 20% as significant (SD, 10%–15%) (14). Using a 20% reduction in SUV60_mean_ to define a PET response led to categorization as lesional response in 60% (15/25) and nonresponse in 40% (10/25). Using Cohen κ-measures, there was good concordance between lesional PET response and lesional mRECIST (κ = 0.66; *P* < 0.001; 95% confidence interval, 0.35–0.97).

### Kinetic Modeling Evidence of Significant Reduction in Uptake and Retention After TACE

The analysis of ^18^F-FLT dynamic data with a 2-tissue-compartment model resulted in physiologically relevant kinetic parameters (*n* = 14) (Table 2) (15). There was a significant mean reduction in the radiotracer delivery parameter *K*_1_ between baseline (0.3 ± 0.1 mL/min/g) and after treatment (0.13 ± 0.05 mL/min/g) (*P* < 0.001). This reduction is consistent with the abrupt cessation of blood flow to the tumor after embolization, resulting in reduced transport of ^18^F-FLT to the tumor. Although all tumors showed some degree of reduction in *K*_1_, the change was greater in responders (66%) than in nonresponders (50%) (*P* = 0.03) (Supplemental Fig. 3A). Baseline SUV60_mean_ and metabolic flux constant (*K*_i_) were significantly correlated (Pearson *r* = 0.5, *P* = 0.04), and a significant difference in *K*_i_ was observed between baseline imaging (0.09 ± 0.03 mL/min/g) and post-TACE imaging (0.04 ± 0.02 mL/min/g) (*P* < 0.001). Baseline *K*_i_ and fractional blood volume (V_b_) were greater in responders than in nonresponders (Supplemental Figs. 3B and 3C) (*P* < 0.05).

**TABLE 2:** Baseline Dynamic PET Parameters (*n* = 14)

Patient no.	*K*_1_ (mL/min/g)	*k*_2_ (1/min)	*k*_3_ (1/min)	*k*_4_ (1/min)	V_b_ (mL/g)	*K*_i_ (mL/min/g)
1	0.16	0.10	0.10	0.11	5.0E−0	0.08
2	0.17	0.076	0.074	0.07	0.015	0.09
3	0.31	0.40	0.24	0.04	0.019	0.11
4	0.21	0.28	0.13	0.06	0.034	0.07
5	0.34	0.17	0.097	0.04	0.03	0.13
6	0.43	0.28	0.086	0.01	0.058	0.10
8	0.282	0.28	0.13	0.02	0.04	0.09
9	0.29	0.53	0.32	0.04	0.05	0.11
11	0.41	0.51	0.17	0.02	0.07	0.10
13	0.11	0.16	0.04	0.001	0.10	0.02
14	0.46	0.42	0.13	0.03	2.9E−04	0.11
15	0.58	0.69	0.13	0.02	0.07	0.09
16	0.26	0.18	0.08	0.02	0.07	0.08
18	0.23	0.19	0.09	0.02	8.0E−06	0.07

## DISCUSSION

There is still no single tracer recommended by international guidelines for either diagnosis or response assessment in HCC (1). The main limitation of the studied tracers has been a poor tumor-to-background liver ratio resulting in a dual-tracer approach for visualizing HCC, which is time-consuming and exposes patients to significant radiation (5). We hypothesized that because ^18^F-FLT uptake is specific for tumor proliferation, tracer uptake will not be affected by the presence of inflammation (16). Moreover, we investigated the utility of KSF to improve visualization of HCC by removing background liver activity.

To address the hypothesis that tumor ^18^F-FLT uptake will change predictably with effective treatment, we first assessed the messenger RNA expression of factors responsible for handling ^18^F-FLT. *TYMS* catalyzes the last step in the de novo synthesis of thymidine monophosphate (TMP), whereas *TK-1* catalyzes synthesis of TMP via the salvage pathway. *TK-1* affects ^18^F-FLT cellular trapping and is a surrogate marker of proliferation (17). Moreover, we have shown that with *TYMS* inhibition, ^18^F-FLT uptake increases because of redistribution of the membrane transporter *SLC29A1* to the plasma membrane (18). Expression levels of *TYMS, TK-1,* and *SLC29A1* were all markedly upregulated in HCC compared with normal liver; we confirmed marked upregulation in protein expression of *TK-1* in HCC compared with surrounding matched cirrhotic tissue consisting of both regenerative and dysplastic nodules, suggesting that ^18^F-FLT could be useful in differentiating HCC from surrounding cirrhotic tissue.

Our prospective ^18^F-FLT PET study illustrated that most intrahepatic lesions had increased tracer uptake consistent with the tissue microarray findings. To improve HCC visualization, we applied the previously validated KSF (19,20). However, fewer lesions were detected with KSF than with standard PET/CT imaging; most small lesions—those smaller than 3 cm—were filtered out. One possible explanation is that the KSF compares voxel temporal profiles with standard tissue profiles and that both liver and lesion voxel profiles were highly variable in our group. Another explanation is that the cirrhotic background has a high relative uptake that, in combination with the profile variability, reduces the ability of the KSF to effectively discriminate HCC from the proliferating, background, liver. A third possible explanation is that partial-volume effects may contribute to filtering out of small lesions.

When considering ^18^F-FLT PET imaging for lesion detection, our findings are in keeping with those of Eckel et al., who reported a 72% sensitivity for ^18^F-FLT in visualizing HCC, with a similar median SUV and tumor-to-liver ratios (10). Similar sensitivities have been reported for ^11^C-acetate PET and for a dual-tracer approach with ^18^F-FDG and ^11^C-acetate, 75% and 73%, respectively (21,22). However, Ho et al. reported single-tracer sensitivity of 87% for ^11^C-acetate, increasing to 100% sensitivity using 2 tracers (23). These differences in diagnostic sensitivity may be a reflection of the subgroup analysis undertaken in the study of Ho et al. Overall, the fact that the literature does suggest improved diagnostic sensitivity using a dual-tracer approach motivates the development of alternate tracers for the detection of HCC.

We investigated the role of dynamic ^18^F-FLT PET as a predictor of TACE response. The radiologic response to TACE was 54% by mRECIST and 60% by PET, with good concordance between imaging modalities. Cascales-Campos et al. considered using ^18^F-FDG PET to assess response to TACE before liver transplantation (24). The authors described a reduction in ^18^F-FDG uptake correlating with the degree of necrosis on pathologic examination of the explanted liver, and other investigators have considered minimum SUV cutoffs for defining response to therapy (25). In a retrospective study, Park et al. investigated the utility of a dual-tracer approach with ^18^F-FDG and ^11^C-acetate in predicting response to TACE (7). They did not investigate concordance between the imaging modalities but observed that the ratio of ^18^F-FDG to ^11^C-acetate predicted response to TACE, determining a cutoff from ROC analysis. We selected a 20% reduction in SUV60_max_ to indicate response, a cutoff extrapolated from breast cancer studies (26). Larger studies are needed to define a more accurate cutoff for HCC than was possible to derive from our small data set.

A key strength of this study is that dynamic PET imaging allows us to establish the basis of the PET signal change in HCC, considering that TACE has an acute impact on blood flow. In line with our hypothesis, we report a significant reduction in *K*_1_ after TACE, illustrating an abrupt reduction in tissue perfusion. This finding is in sharp contrast to antiangiogenic chemotherapy, for which an increase in *K*_1_ due to vessel normalization and reduced interstitial pressure can accompany response (27). Dynamic studies using ^11^C-acetate reported a reduced *K*_1_ in HCC lesions supplied by the hepatic artery, compared with benign lesions supplied by the portal vein, because the radiotracer concentration time-course is initially delayed as the portal flow passes through the splanchnic circulation (28). Huo et al. reported that because of the differential time course of radiotracers circulating through the hepatic artery or portal vein, lesions supplied by the hepatic artery will reach a stable concentration of radiotracer earlier and at a higher peak after injection; hence, arterialized lesions will have a lower *K*_1_ than benign lesions that are supplied predominantly by the portal vein (29). *K*_i_ is related to the phosphorylation of thymidine in the tissue (30). *K*_i_ has previously been found to correlate with ^18^F-FLT SUV (31), and our results illustrate a similar correlation. Moreover, we report baseline *K*_i_ and V_b_ to be predictive of TACE response to treatment, suggesting that responding tumors are more actively proliferating and have higher perfusion suggestive of higher vascularity.

Study limitations include a small sample size and a lack of correlation between PET uptake parameters and histology. If subjects have whole-body imaging, full assessment of extrahepatic disease could be performed; the correlation between PET kinetic parameters and SUV means that future whole-body static imaging would be supported. In addition, manual VOIs could not be used to determine thresholds because components of the tumor were close to background liver activity. In addition, some lesions became isointense or photopenic relative to background on follow-up PET imaging.

## CONCLUSION

We have shown that *TK-1* expression is significantly higher in HCC than in surrounding cirrhotic tissue, supporting the use of ^18^F-FLT PET. High ^18^F-FLT PET uptake was seen in most HCC tumors; however, application of KSF did not improve visualization because of the high and variable SUV both in tumors and in background cirrhotic liver. Imaging of proliferation with ^18^F-FLT PET could be used to predict response to TACE in this small case series. Although this study was a pilot study, the results generated are provocative and should be taken forward to larger prospective trials correlating with outcome.

## DISCLOSURE

This study was supported by the Imperial College NIHR Imperial Biomedical Research Centre and the Imperial Experimental Cancer Medicines Centre, Cancer Research U.K. (C2536/A16584). The views expressed are those of the authors and not necessarily those of the NIHR or the Department of Health and Social Care. No other potential conflict of interest relevant to this article was reported.

**KEY POINTS**

**QUESTION:** Does dynamic ^18^F-FLT PET allow accurate visualization of HCC?
**PERTINENT FINDINGS:** ^18^F-FLT PET can differentiate HCC from surrounding cirrhotic tissue, with PET parameters correlating with TACE response.
**IMPLICATIONS FOR PATIENT CARE:** ^18^F-FLT PET can accurately detect HCC and should be further investigated, particularly for assessment of response.
